# Early Visual Attention in Preterm and Fullterm Infants in Relation to Cognitive and Motor Outcomes at School Age: An Exploratory Study

**DOI:** 10.3389/fped.2014.00106

**Published:** 2014-10-06

**Authors:** Marrit M. Hitzert, Koenraad N. J. A. Van Braeckel, Arend F. Bos, Sabine Hunnius, Reint H. Geuze

**Affiliations:** ^1^Division of Neonatology, Department of Pediatrics, Beatrix Children’s Hospital, University Medical Center Groningen, University of Groningen, Groningen, Netherlands; ^2^Donders Institute for Brain, Cognition and Behaviour, Radboud University Nijmegen, Nijmegen, Netherlands; ^3^Department of Clinical and Developmental Neuropsychology, University of Groningen, Groningen, Netherlands

**Keywords:** frequency of looks, response latencies, visual competing stimuli, motor skills, cognition, functional development, low-risk preterm infants, longitudinal study

## Abstract

**Objective:** Preterm infants are exposed to the visual environment earlier than fullterm infants, but whether early exposure affects later development is unclear. Our aim was to investigate whether the development of visual disengagement capacity during the first 6 months postterm was associated with cognitive and motor outcomes at school age, and whether associations differed between fullterms and low-risk preterms.

**Method:** Seventeen fullterms and ten low-risk preterms were tested in a gaze shifting task every 4 weeks until 6 months postterm. The longitudinal data were converted into single continuous variables by fitting the data with an S-shaped curve (frequencies of looks) or an inverse model (latencies of looks). Neuropsychological test results at school age were converted into composite *z* scores. We then performed linear regression analyses for each functional domain at school age with the variables measuring infant visual attention as separate predictors and adjusting for maternal level of education and group (fullterms versus preterms). We included an interaction term, visual attention*group, to determine whether predictive relations differed between fullterms and preterms.

**Results:** A slower development of disengagement predicted poorer performance on attention, motor skills, and handwriting, irrespective of fullterm or preterm birth. Predictive relationships differed marginally between fullterms and preterms for inhibitory attentional control (*P* = 0.054) and comprehensive reading (*P* = 0.064).

**Conclusion:** This exploratory study yielded no indications of a clear advantage or disadvantage of the extra visual exposure in healthy preterm infants. We tentatively conclude that additional visual exposure does not interfere with the ongoing development of neuronal networks during this vulnerable period of brain development.

## Introduction

During the first half year of life, looking is one of the most important behavior young infants have to explore their surroundings (1). Sensory-motor processes involved in detecting and shifting gaze to visual targets are already functional as early as 40 weeks of gestation (2). Between the ages of approximately 1 and 3 months, however, infants experience difficulties particularly in shifting gaze from a persistent stimulus in the center of their visual field to a stimulus in the periphery, thus under competitive conditions requiring disengagement of attention (3, 4). The frequency and speed of shifting gaze under competitive conditions increase substantially around 3 to 4 months of age (2, 4), but it is not before 5 to 6 months that this ability reaches adult levels (5, 6). Evidence is accumulating that the increased ability of infants to shift gaze from one location to another not only enhances the visual exploration of the environment but also forms the basis for social interaction and self-regulation, skills which are fundamental to cognitive development. For instance, in a cohort of fullterm-born infants, recognition memory measured with novelty scores from a paired-comparison task at 7 months predicted intelligence and academic achievement at the age of 21 years (7). In preterms, longer gaze fixations at term age are related to poorer focused attention and lower intelligence at 12 years of age (8). For an overview see Hunnius et al. (9).

The past decades have shown growing interest in the development of visual attention and the associated development of the brain (10–14). Visual attention can be studied in infants by observing gaze shifts under different circumstances. According to Atkinson et al. (15), disengaging attention and switching gaze during the first 6 months of life is subserved by two attention networks in the brain: (1) subcortical systems involving the superior colliculus underlie the ability to shift fixation from a central target to a salient peripheral target, provided both targets are not visible together and without other visual or auditory “distracters” in the rest of the visual field, and (2) cortical systems underlie disengaging attention and gaze from an object or stimulus that is currently fixated. Both systems are closely interconnected with the extended occipital and posterior parietal or dorsal visual stream of visual–spatial processing (16–18). Disengaging attention and gaze from current focus is thought to be mediated by the posterior parietal cortex (including the intraparietal sulcus) and frontal cortex (including frontal eye fields). The superior colliculus is thought to be involved in shifting the gaze to a new location and inhibiting a location already attended to. Reengaging attention at the new location is thought to be mediated by the thalamus. For a review, see Petersen and Posner (14).

Various studies reported that the developmental trajectory of visual attention of preterm and fullterm-born infants differs during the first 6 months of life. High-risk preterms have longer look durations, slower disengagement, and attention shifts, and they shift less between stimuli than their fullterm-born peers (19–21). In low-risk preterms, however, the rates of gaze shifts are temporarily faster than those of fullterms (22–25). Hunnius and colleagues attributed this finding to the fact that the additional visual exposure experienced by preterms in comparison to their fullterm peers may have accelerated the maturation of cortical processes involved in disengaging (25). An explanation for the differences observed between high-risk and low-risk preterms might be that the findings in the former stem mainly from perinatal complications and brain damage rather than reflecting the supposed effect of additional visual experience on the development of early visual attention. This is in line with the poor performance on gaze shifting tasks as an indicator of the location or extent of cerebral injury (26–30). We found 3 longitudinal studies that examined whether the association between early visual attention measures and later IQ differed between fullterms and preterms (31–35). One study revealed that better performance on visual habituation and visual recognition memory tasks was more strongly associated with higher IQ at the age of 2 to 5 years in preterm-born children than in fullterm-born children (35). Rose and colleagues, however, were unable to demonstrate a difference in the predictive value of visual attention measures for IQ up to 11 years of age, neither between high-risk preterms and fullterms (32), nor between low-risk preterms and fullterms (34).

It is striking that most studies focused on later intelligence, attention skills, or academic achievement. Considering the strong link between attention networks and the dorsal visual stream, early visual attention might also be related to other functions closely associated with the dorsal stream, such as visuomotor coordination, spatial cognition, and executive functioning (15, 36–38). Not only is early visual attention considered pivotal in the development of higher cognitive functions, it may also play a role in the development of motor skills since dorsal-stream information feeds into systems used during visual–spatial manipulation and visual control of action (39).

To date, the question whether gaze shifting, as a marker of early visual attention, is related to specific cognitive functions and complex motor skills at school age has not been investigated. Additionally, it remains unclear whether the observed differences in visual attention between low-risk preterms and fullterms are linked to specific deficits at school age. If the accelerated maturation of visual attention in low-risk preterms interferes with the ongoing development of related neuronal networks, this might eventually lead to poorer performance at school age. In the literature, we did find a report that visual attention markers, such as infant habituation and recognition memory, which serve as predictors of later IQ, are strongest in infant assessments made between approximately 10 and 18 weeks (40). Furthermore, changes in attentional functions measured longitudinally during periods of rapid development might be better indicators of early cognitive functioning than attentional function measurements limited to one age (3). To the best of our knowledge, no studies have examined the predictive value of gaze shifts under both competitive and non-competitive conditions for functioning at school age.

The aim of our study was, therefore, to investigate whether the development of gaze shifts toward a peripheral stimulus during the first 6 months was associated with specific cognitive functions and complex motor skills at school age, and to determine whether these associations differed between fullterms and preterms. We examined gaze shifts under competitive and non-competitive conditions, since gaze shifts under these two conditions are thought to measure two distinct processes, i.e., visuomotor processes and additional attentional processes, respectively.

Given the close connection between attention networks and the dorsal stream of cortical visual–spatial processing, we expected to find in both the competitive and non-competitive trials that a slower development of gaze shifts toward adult levels might be related to poorer cognitive functions and poorer motor skills. Most evidence for the relation between early visual attention and later cognitive performance was provided by studies on look durations (inability to disengage) or visual recognition memory (looking away from a familiar stimulus to a novel stimulus). These studies suggested that differences in the development of early visual attention lie at the basis of differences in cognitive abilities later. We therefore expected to find the strongest associations for performance on competitive rather than non-competitive trials since the latter do not require disengagement. Moreover, since the competitive trials are supposedly more challenging, we expected performance on these trials to have a higher discriminatory potential for later development than performance on the non-competitive trials.

For this study, we collected follow-up data on cognitive and motor functioning at school age for a group of fullterm and preterm children who as infants had been tested on a visual attention task (4, 25).

## Materials and Methods

### Participants

Our study population consisted of fullterm and preterm infants who had formerly been included in a longitudinal study on the development of visual attention (25). The fullterm group consisted of 20 infants whose mothers had been approached through childbirth education classes, midwives, or gym classes during 2000–2002. Exclusion criteria were <37 or >42 weeks’ gestation, a birth weight below 2800 g, and a history of prenatal and/or perinatal complications. The preterm group consisted of 10 infants born at <32 weeks’ gestation. These infants had been admitted to University Medical Center Groningen between 2000 and 2002. Exclusion criteria were risk factors for abnormal neurological development, including >14 days of ventilation, severe hemorrhagic and ischemic brain lesions, and serious infections. Infants with retinopathy of prematurity of >Stage I were also excluded.

Two families declined the invitation to participate. One child in the fullterm group had moved abroad and could not be tested. All parents of the preterm group agreed to their children participating in the study. We present the perinatal demographics in Table 1. In Table 2, the characteristics at follow-up are presented.

**Table 1:** **Perinatal characteristics of the fullterm and preterm group**.

	Fullterms (*n* = 17)	Preterms (*n* = 10)
Gender (boys/girls)	6/11	6/4
Gestational age (weeks)	40.3 (37.0–42.0)	29.2 (27.3– 32.0)
Birth weight (g)	3550 (2880–4100)	1130 (640– 2035)
IUGR (<10th percentile)	0 (0%)	1 (10%)
Apgar score at 5 min		8 (1– 9)
Asphyxia		5 (50%)
Late-onset sepsis (positive blood culture)		1 (10%)
Retinopathy of prematurity^a^		0 (0%)
BPD (O_2_ at 36 weeks’ PMA)		4 (40%)
Mechanical ventilation (days)		4 (1– 13)
Cerebral pathology
None		3 (30%)
PVE > 7 days		3 (30%)
Mild^b^		4 (40%)
Severe^c^		0 (0%)

*Data are given as median (minimum–maximum) or numbers (percentage). IUGR, intra-uterine growth restriction; BPD, bronchopulmonary dysplasia; PMA, postmenstrual age; PVE, periventricular echodensities. Cranial ultrasound results were graded according to Papile et al. (41) and de Vries et al. (42). Empty boxes indicate that data were not available or did not apply*.

*^a^Retinopathy of prematurity Stage II or worse*.

*^b^Mild cerebral pathology was defined as Grades I and II germinal matrix–intraventricular hemorrhage (GMH–IVH)*.

*^c^Severe cerebral pathology was defined as Grade III GMH-IVH, periventricular hemorrhagic infarction, posthemorrhagic ventricular dilatation (PHVD), and cystic periventricular leukomalacia. PHVD was defined as a lateral ventricle size of >0.33 according to Evans’ index*.

**Table 2:** **Characteristics of the fullterm and preterm group at follow-up**.

	Fullterms (*n* = 17)	Preterms (*n* = 10)
Age at follow-up (years, months)	11.0 (9.9−11.8)	10.5 (9.8− 11.4)
Special education	0 (0%)	2 (20%)
Glasses	4 (24%)	2 (20%)
Maternal level of education
≤11 years	1 (6%)	1 (10%)
12-13 years	2 (12%)	6 (60%)
≥14 years	14 (82%)	3 (30%)

*Data are given as median (minimum–maximum) or numbers (percentage)*.

### Measurement of visual attention during the first 6 months

Measurement sessions were conducted at 6, 10, 14, 18, 22, and 26 weeks, calculated from the due date. Infants were tested in a gaze shifting task consisting of competitive trials (*n* = 32) and non-competitive trials (*n* = 8). All trials started with the appearance of a stimulus in the center of the computer screen. After the infant had fixated the central stimulus for 1–2 s, a second stimulus was displayed in the periphery. While during non-competitive trials the central stimulus disappeared followed by a peripheral target, during competitive trials the central stimulus remained visible after the peripheral target had appeared. Frequencies and latencies of gaze shifts toward the peripheral stimulus under non-competitive conditions provide an index of the efficiency of visuomotor processing involved in detecting the new target, and of preparing and executing an eye movement toward the peripheral target. Competitive trials require disengagement from the attended stimulus before an eye movement is made to the peripheral target. Frequencies and latencies of gaze shifts under competitive conditions thus provide an index of attentional processes in addition to visuomotor processes. A detailed description of the testing situation and the coding of eye movements is provided by Hunnius et al. (25).

### Follow-up

When participants were 9 to 11 years old, we assessed cognitive, motor, and visual functions in detail. See Table 3 for a description of the tests and questionnaires. Parents gave their written informed consent prior to their infants’ participation in the follow-up program. The study was approved by the Medical Ethics Committee of University Medical Center Groningen.

**Table 3:** **Measurements, related motor, and cognitive functions, referring names in the text, and assigned domains**.

Domain	Test/scale	Function	Referring name
Intelligence	WISC-III Total IQ	Total intelligence	TIQ
	WISC-III Verbal IQ	Verbal intelligence	VIQ
	WISC-III Performance IQ	Performance intelligence	PIQ
Attention	TEA-Ch-NL Map mission	Selective visual attention	Selective attention
	TEA-Ch-NL Opposite world	Inhibition of automatic response	Inhibition
Visuomotor	NEPSY-II Design copying	Visuomotor functioning	Visuomotor
Visual-spatial perception	NEPSY-II Picture puzzles	Visual discrimination and visual scanning	Picture puzzles
	NEPSY-II Arrows	Visual-spatial processing	Arrows
	NEPSY-II Route finding	Visual-spatial relations, orientation, and directionality	Route finding
Visual object perception	TVPS-3 Form constancy	Visual perception: form constancy	Form constancy
	TVPS-3 Visual closure	Visual perception: visual closure	Visual closure
	TVPS-3 Form discrimination	Visual perception: form discrimination	Form discrimination
Executive functioning	BRIEF Global executive composition	Well-organized, purposeful, goal-directed, and problem-solving behavior	GEC
Academic achievement	Cito mathematics	Standardized Dutch scholastic achievement test – subscale mathematics	Mathematics
	Cito spelling	Standardized Dutch scholastic achievement test – subscale spelling	Spelling
	Cito comprehensive reading	Standardized Dutch scholastic achievement test – subscale comprehensive reading	Comprehensive reading
	Cito technical reading	Standardized Dutch scholastic achievement test – subscale technical reading	Technical reading
Motor skill	Movement-ABC total	Motor proficiency of everyday motor skills	Motor skill
	Fine motor	Manual dexterity	Manual dexterity
	Ball skills	Object control	Object control
	Balance	Postural control	Postural control
	BHK	Handwriting	Handwriting
	DCD-Q	Motor problems in daily life	Motor problems

*WISC-III-NL, shortened form of the Wechsler Intelligence Scale for Children, third edition, Dutch version (43, 44); TEACh-NL, Test of Everyday Attention for Children (45); NEPSY-II, Neuropsychological Assessment, second edition (46); TVPS-3, Test of Visual–Perceptual Skills, third edition (47); BRIEF, Behavior Rating Inventory of Executive Function (48); GEC, global executive functioning; Cito, Centraal Instituut voor Toetsontwikkeling (a Dutch scholastic achievement test); Movement-ABC, Movement Assessment Battery for Children (49); BHK, Beknopte beoordelingsmethode voor Handschriften van Kinderen (Concise Assessment Scale for Children’s Handwriting) (50); DCD-Q, Developmental Coordination Disorder Questionnaire (51)*.

### Motor and cognitive outcomes

Motor outcome was assessed using the Movement Assessment Battery for Children (Movement-ABC) (49), a standardized test of motor skills for children 4 to 12 years of age. This test, which is widely used in practice and in research, yields a score for total movement performance based on separate scores for manual dexterity (fine motor skills), object control (ball skills), and postural control (balance). Handwriting was tested with the Concise Assessment Scale for Children’s Handwriting (BHK) (50). The handwriting test consists of copying a standard text for 5 min on an A4 size, unlined sheet of paper. Quality was measured according to 13 features. We used the Dutch version of the Developmental Coordination Disorder Questionnaire (DCD-Q) to screen for motor problems in daily life (51). This questionnaire, which is filled out by the parents, was developed to identify motor problems in children >4 years of age. It contains 17 items relating to motor coordination, which are classified into 3 categories: control during movement, fine motor skills/writing, and general coordination.

Total, verbal, and performance intelligence were assessed using a shortened form of the Wechsler Intelligence Scale for Children, third edition, Dutch version (WISC-III) (43, 44). Examples on items of the WISC-III are vocabulary, analogies, organizing pictures, and reproduction of block designs. We measured selective attention and attentional control with the subtests Map mission and Opposite worlds of the Test of Everyday Attention for Children (TEA-Ch) (45). Selective attention refers to the ability to select target information from an array of distractors. For example, the child was asked to select target symbols from an array of distractor symbols. In the attentional control task, the child is asked to name a set of numbers (i.e., alternating numbers 1 and 2). In the second task, the child is asked to name the opposite of what is shown (i.e., 1 instead of 2 and vice versa). We assessed visuomotor integration with the Design copying subtest of the NEPSY-II (Neuropsychological Assessment, second edition) (46). In this subtest, the child is asked to reproduce geometric forms of increasing complexity. Visuomotor integration involves the integration of visual information with finger–hand movements. Visual–spatial perception was assessed by 3 subtests of the NEPSY-II. In the subtest Picture puzzles, the child is presented a large picture divided by a grid and four smaller pictures taken from sections of the larger picture. The child has to identify the location on the grid of the larger picture from which each of the smaller pictures was taken. In the subtest Arrows, the child looks at an array of arrows arranged around a target and indicates the arrow(s) that points to the center of the target. In the subtest Route finding, the child is shown a schematic map with a target house and asked to find that house in a larger map with other houses and streets. Visual object perception was measured with 3 subtests of the Test of Visual-Perceptual Skills, third edition (TVPS-3) (47). In the Form constancy task, the child is asked to find one design among others on the page; the design can be larger, smaller, or rotated. In the Visual closure task, the child is shown a completed design on the page and is asked to match it to one of the incomplete patterns shown on the page. In the last subtest, Form discrimination, the child is shown a design and is asked to point to the matching design among the choices shown on the page. We obtained information on children’s executive functioning involved in well-organized, purposeful, goal-directed, and problem-solving behavior by using the Behavior Rating Inventory of Executive Function (BRIEF) questionnaire (48), which was filled out by the parents. The BRIEF contains 75 items in 8 non-overlapping clinical scales that form 2 broader indexes: behavioral regulation (inhibit, shift, and emotional control subscales) and metacognition (initiate, working memory, plan/organize, organization, and monitor subscales). Together these scales form the Global Executive Composite (GEC) score, which represents the child’s overall executive functioning.

The total duration of the follow-up was approximately 3 h including breaks. Test scores obtained when a child was too tired, as assessed by the trained experimenter, were excluded.

We sought permission from the parents to contact their children’s schools for their most recent results on the so-called Cito test for mathematics, spelling, comprehensive reading, and technical reading skills. Cito, which stands for Central Institute for Test Development, is a standardized Dutch scholastic achievement test conducted twice annually at primary schools – in the middle and at the end of the school year (for interpretation guidelines of the standard Cito scores see: http://www.cito.nl; retrieved on December 17th, 2013). The Cito scores are expressed in levels of performance: Level I represents the 20% of children with the highest scores and Level V represents the 20% of children with the lowest scores.

### Vision

Vision was defined according to the 10th revision of the International Statistical Classification of Diseases (ICD-10): mild or no visual impairment if visual acuity was ≥0.3; moderate visual impairment if visual acuity was between 0.1 and 0.3; severe visual impairment if visual acuity was between 0.05 and 0.1; blindness if visual acuity was <0.05 or if there was no light perception (52). Visual acuity was tested with the Landolt C chart (correction with prescription glasses allowed) and visual field with Donders’ method.

### Statistical analyses

For the infancy data, we calculated the relative frequency of looks (frequency of looks divided by the number of trials), and the median latencies between appearance of the peripheral stimulus and the onset of an eye movement toward the target. The frequency of looks represents the ability to shift the gaze toward a peripheral stimulus. The latencies of gaze shifts represent the speed of disengaging and shifting the gaze toward a peripheral stimulus. For the analyses of gaze shifting latencies, the first measurement at 6 weeks was excluded because shifts of gaze were very infrequent, and therefore only few data points were available.

To relate the longitudinal data of the disengagement tasks with cognitive and motor outcomes at school age, we converted the longitudinal data of the disengagement tasks into single continuous variables, for the competitive and non-competitive conditions separately.

For the frequency of looks, we determined the age at which the infant reached a relative frequency of looks of 50% by least square fitting the data with an S-shaped curve for the interval 0–1

$$y\left(t\right)=\frac{L_{\mathrm{end}}}{\left(1+\frac{\left(L_{\mathrm{end}}-L_{\mathrm{begin}}\right)}{L_{\mathrm{begin}}}\right)\times e^{-c\times t}}$$

with *t* being the age in weeks, *L*_end_ being the maximum relative frequency of looks (i.e., 1.0), *L*_begin_ being the minimum of relative frequency of looks (set to 0.01), and *c* being a constant that determined the growth rate or steepness of the S-curve. For each individual set of longitudinal data, *c* was varied by iteration to reach an optimal least squares fit. Throughout this article this variable is referred to as 50%-looks.

For the latencies of looks [reaction time (RT)], we used the inverse model *y*(*t*) = *b*_0_ + *b*_1_/*t* to fit the data. Variable *b*_0_ represents the final level of RT reached due to development (i.e., adult level); *b*_1_ represents the rate of change toward that level. A higher *b*_1_ value reflects a slower development toward the adult level of RT (*b*_0_). We set *b*_0_ at 200 ms, based on the assumption that the adult value of RT (*b*_0_) approaches 200 ms (53). In the analyses we used the variable *b*_1_. Throughout this article, this variable is referred to as b1-RT. The variable b1-RT was calculated separately for competitive and non-competitive trials. Altogether we derived four infancy measures of visual attention: 50% looks competitive, 50%-looks non-competitive, b1-RT competitive, and B1-RT non-competitive.

The neuropsychological test results at school age were converted to *z* scores based on the norm scores and percentiles given in the test manuals. The composite scores on each domain were calculated by averaging the *z* scores of the subtests as indicated in Table 3. The composite scores on motor skills were calculated by averaging the *z* scores for Movement-ABC Total and the *z* scores for DCD-Q. The *z* scores on the Cito and handwriting test, BHK (Table 3), could not be calculated due to the lack of standardized scores. For BHK, we classified raw scores into non-dysgraphia, borderline, or dysgraphia in accordance with the criteria in the manual. The BRIEF and DCD-Q questionnaires of one preterm child had not been submitted. We replaced the missing composite scores on the executive functioning and motor skills domains by the mean composite score of the preterm group on these domains. We did not correct for age at follow-up in the further analyses since the scores were derived from age-adjusted norms.

First, we analyzed whether our independent variables (visual attention markers) and dependent variables (composite outcome scores) differed between fullterms and preterms. For continuous data, we used the independent-samples Student *t* test in case of normality and the Mann–Whitney *U* test in case of non-normality. For categorical data, we used Fisher’s exact test. We controlled for mothers’ level of education when comparing cognitive and motor outcomes between fullterms and preterms, since SES may act as a confounding variable (54).

The first question we addressed was whether the development of gaze shifts toward a peripheral stimulus during the first 6 months was associated with specific cognitive functions and complex motor skills at school age. We performed univariate linear regression analyses for each school age outcome composite score with the variables measuring infant visual attention as separate predictors. Next, we analyzed each of the subtests of the composite scores to determine which subtest contributed most to the predictive relation, but only if the *P* value of that composite score was below 0.15 to limit multiple testing. Thus, if none of the visual attention predictors were associated with the composite outcome score (*P* > 0.15) we did not repeat the analyses for the subtests comprising the composite score. We controlled for mothers’ education and group. The former was entered as a nominal predictor (low and average versus high educational level) since only one mother (of a fullterm child) had a low educational level. Since we had no *z* scores on Cito and BHK, we performed logistic regression for these outcomes instead (Cito Levels IV or V considered abnormal; BHK borderline and dysgraphia considered abnormal). Additionally, we determined the predictive value of visual attention markers for overall functioning at school age (cognitive and motor outcomes combined). For this purpose, multivariate analyses would be the method of first choice. *A priori*, we performed a sample size calculation for multivariate regression with a power of 0.80, an alpha of 0.05, an anticipated effect size of 0.20 (*f*  ^2^), a number of groups of 2 (fullterms and preterms), a number of predictors of 4 (visual attention measures), and a number of response variables of 9 (the 8 domains as given in Table 3 plus handwriting), which yielded a required sample size of 62 infants. Since we were only able to include 27 infants in our study sample, multivariate analyses might provide unreliable results. As an alternative, therefore, we repeated the univariate analyses for the mean composite scores on all cognitive and motor domains as dependent variable (Cito and BHK excluded due to the lack of *z* scores).

The second question we addressed was whether predictive relations of visual attention in infancy for outcomes at school age differed between fullterms and preterms. To answer this question we included an interaction term (visual attention marker*group) in all the regression analyses.

Throughout the analyses *P* < 0.05 was considered statistically significant. We used SPSS 20.0 (SPSS Inc., Chicago, IL, USA) for the analyses. Because 9 outcome measures were tested against 4 hypothesized visual attention predictors, a Bonferroni-adjusted significance level of 0.0014 was calculated to account for the increased possibility of type-I error due to multiple testing.

## Results

### Visual attention during the first 6 months postterm

We provide an overview of the markers of visual attention in Table 4. Of these only RT in the competitive trials differed between fullterms and preterms with the preterms having a faster development toward adult RT (*P* = 0.046).^1^

**Table 4:** **Overview of visual attention markers over sessions 2–6, and *P* values of group differences**.

	Fullterms (*n* = 17)	Preterms (*n* = 10)	*P* value
**Competitive condition**
Frequency of looks (weeks)	14.6 (9.5–19.0)	14.1 (9.9–23.0)	0.824
Reaction time (RT)	9.4 (4.4–16.9)	6.8 (3.5–15.6)	0.046*
**Non-competition**
Frequency of looks (weeks)	6.5 (4.3–10.4)	6.4 (2.6–21.2)	0.902
Reaction time (RT)	3.2 (2.2–5.3)	3.2 (2.3–9.8)	0.711

Values are given as median (minimum–maximum). *P* values were calculated using the Mann–Whitney *U* test. Frequency of looks was defined as the age at which the infant reached 50% of the maximum relative frequency of looks. Reaction time was defined as the speed at which the child grows toward a lower reaction time (b1). A higher b1 value represents a slower development toward a lower reaction time. *P < 0.05.

### Group differences at school age

**Figure 1:**
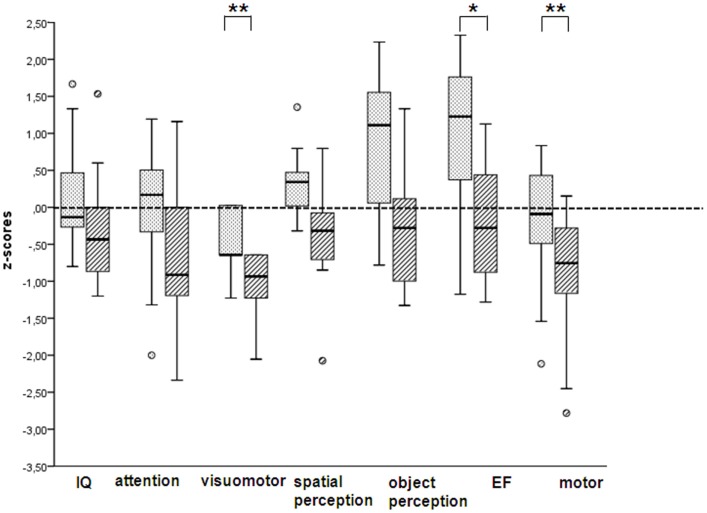
**Composite scores on cognitive and motor outcomes, expressed as *z* scores, in fullterm-born (dotted) and preterm-born children (hatched)**. Data are presented as box and whisker plots. The boxes represent values between the 25th and 75th percentiles. The whiskers represent the range of the values, with the exception of outliers, which are represented as circles. Statistical differences were calculated after controlling for maternal education. IQ, intelligence quotient; EF, executive functioning **P < 0.05; *P < 0.1.

Preterm children had poorer scores on the cognitive and motor tests compared to their fullterm peers (see Supplementary Material). After calculating composite scores, the preterm group had significantly lower *z* scores after controlling for maternal education on the domains (see Figure 1): visuomotor (*B* = −0.534; 95% CI, −0.975 to −0.094; *P* = 0.019) and motor (*B* = −1.007; 95% CI, −1.95 to − 0.060; *P* = 0.038). Preterms scored marginally lower on executive functioning (*B* = −0.744; 95% CI, −1.620 to 0.133; *P* = 0.093). Scores on Cito and BHK did not differ between fullterms and preterms (see Table 5).

**Table 5:** **Scores on the Cito test and handwriting, and the statistical significance of group differences**.

Subtest	Fullterms		Preterms		OR (95% CI)	*P* value
	Normal	Abnormal	Normal	Abnormal	
Cito mathematics	15	2	6	4	1.61 (0.15–17.15)	0.69
Cito spelling	16	1	5	5	7.37 (0.56–96.97)	0.13
Cito comprehensive reading	14	3	6	4	0.53 (0.04–7.47)	0.64
Cito technical reading skills	12	4	6	3	0.30 (0.02–4.28)	0.38
Handwriting	15	2	5	5	4.0 (0.46–34.84)	0.21

*Regarding the Cito subtests, normal outcome was defined as Cito Levels I–III and abnormal outcome as Cito levels IV–V. Regarding handwriting, normal outcome was defined as non-dysgraphia and abnormal outcome as borderline or dysgraphia. *P* values represent statistical differences between the fullterm-born and preterm-born children after controlling for maternal level of education*.

### Relationship between visual attention during the first 6 months postterm and cognitive and motor outcomes at school age

In Table 6, we provide the univariate regression analyses, without interaction terms, predicting the different cognitive and motor domains after controlling for group and maternal education.

**Table 6:** **Univariate and adjusted linear regression analyses for each functional domain at school age**.

Functional domain	Maternal education	50%-looks non-competitive	b1-RT non-competitive	50%-looks competitive	b1-RT competitive
IQ
Univariate	0.667 (0.125–1.208); 0.21**				
Adjusted	–	−0.042 (−0.124–0.041); 0.24	−0.123 (−0.302–0.055); 0.27	−0.052 (−0.136–0.032); 0.26	−0.056 (−0.130–0.019); 0.28*
Attention
Univariate	0.973 (0.247–1.698); 0.23**				
Adjusted	–	−0.060 (−0.170–0.050); 0.28	−0.163 (−0.402–0.075); 0.30	−0.067 (−0.179–0.046); 0.28	−0.102 (−0.197–0.008); 0.37**
Visuomotor
Univariate	0.369 (−0.043–0.781); 0.12*				
Adjusted	–	0.010 (−0.047–0.067); 0.31	−0.010 (−0.136–0.116); 0.30	0.00 (−0.059–0.059); 0.30	−0.024 (−0.076–0.028); 033
Visuospatial
Univariate	0.611 (0.127–1.096); 0.21**				
Adjusted	–	−0.025 (−0.097–0.047); 0.29	−0.108 (−0.261–0.044); 0.34	−0.002 (−0.076–0.073); 0.28	−0.031 (−0.097–0.034); 0.31
Visual perception
Univariate	0.968 (0.214–1.722); 0.22**				
Adjusted	–	−0.018 (−0.129–0.093); 0.30	−0.084 (−0.327–0.159); 0.31	−0.004 (−0.118–0.111); 0.30	−0.038 (−0.139–0.064); 0.32
Executive functioning
Univariate	1.018 (0.242–1.793); 0.23**				
Adjusted	–	0.010 (−0.104–0.124); 0.32	0.009 (−0.242–0.260); 0.31	0.027 (−0.090–0.144); 0.32	−0.021 (−0.126–0.084); 0.32
Motor
Univariate	−0.365 (−1.229–0.499); 0.03				
Adjusted	-	−0.078 (−0.197–0.040); 0.25	−0.193 (−0.451–0.065); 0.27*	−0.056 (−0.180–0.068); 0.22	0.038 (−0.074–0.151); 0.21

*Data are displayed as B (95% CI); *R*^2^. Regression analyses were adjusted for group and maternal education. Group was defined as fullterm (reference category) or preterm. Maternal education was defined as low or average (reference category) or high. Looks was defined as the age at which the infant reached a relative frequency of looks of 50%. Reaction time was defined as the speed at which the child grows toward a lower RT (b1). A higher b1 value represents a slower development toward a lower RT*.

*P < 0.15.

**P < 0.05.

The maternal level of education was significantly associated with outcome on the domains: IQ (*B* = 0.667; 95% CI, 0.125–1.208; *R*^2^ = 0.21; *P* = 0.018), attention (*B* = 0.973; 95% CI, 0.247–1.698; *R*^2^ = 0.23; *P* = 0.011), visual–spatial (*B* = 0.611; 95% CI, 0.127–1.096; *R*^2^ = 0.21; *P* = 0.015), visual perception (*B* = 0.968; 95% CI, 0.214–1.722; *R*^2^ = 0.22; *P* = 0.014), and executive functioning (*B* = 1.018; 95% CI, 0.242–1.793; *R*^2^ = 0.23; *P* = 0.012). After applying Bonferroni corrections, none of the associations reached statistical significance.

**Figure 2:**
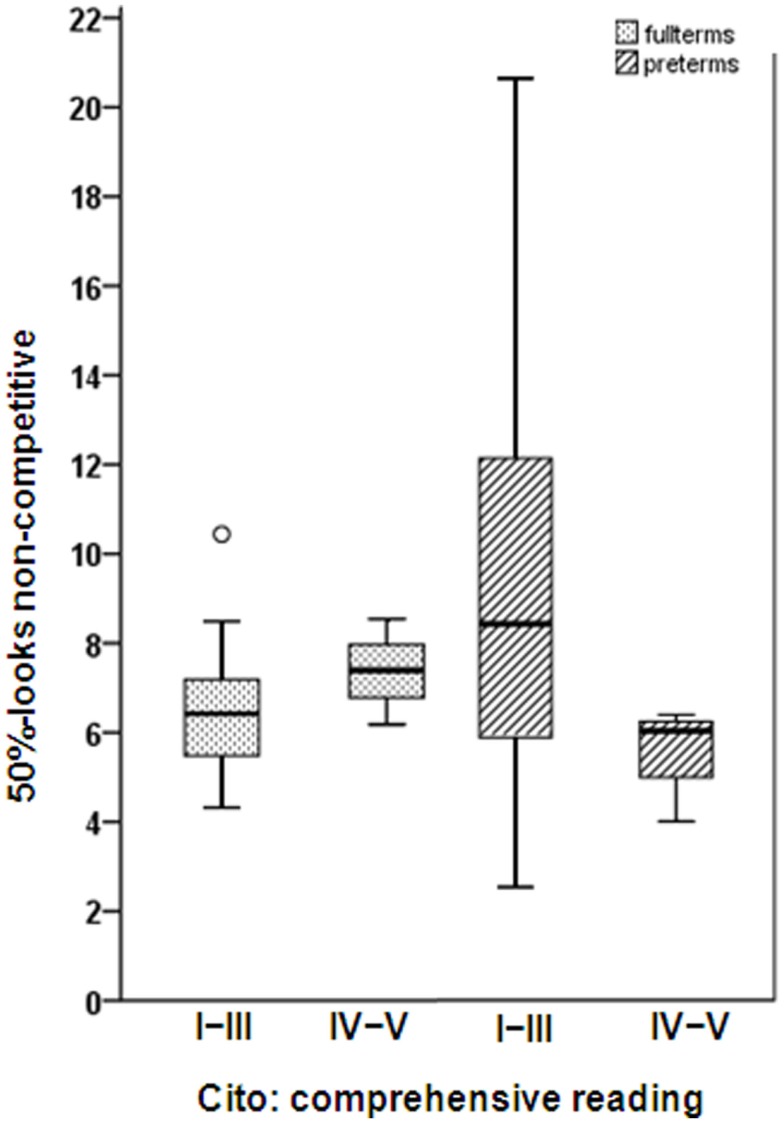
**Age in weeks at which the infant reached a relative frequency of looks of 50% (50%-looks) under the non-competitive condition in fullterm-born (dotted) and preterm-born children (hatched) with normal (Levels I–III) and abnormal (Levels IV–V) scores on the Cito comprehensive reading test**. The data in the graphs are presented as box and whisker plots. Boxes represent the individual values between the 25th and 75th centiles (interquartile range); whiskers represent the range of the values, with the exception of outliers. The outliers are represented by the circles and defined as values between 1.5 interquartile range and 3 interquartile ranges from the end of a box.

For non-competitive conditions, a slower attainment of 50%-looks was marginally associated with poorer handwriting (OR = 2.00; 95% CI, 0.926–4.330; *R*^2^ = 0.607; *P* = 0.077; not shown). Adding the interaction term 50%-looks non-competitive*group revealed that the predictive relation of 50%-looks for comprehensive reading at school age differed marginally between fullterms and preterms. A slower attainment of 50%-looks tended to be associated with better scores on comprehensive reading in the preterms but with poorer scores for the fullterms (looks non-competitive *B* = 0.932; 95% CI, 0.750–8.600; *R*^2^ = 0.47; *P* = 0.134 and looks non-competitive*group *B* = −1.959; 95% CI, 0.018–1.118; *R*^2^ = 0.47; *P* = 0.064; see Figure 2). Regarding the latencies of looks (b1-RT), we found no significant association with motor skills at school age (*B* = −0.193; 95% CI, −0.451–0.065; *R*^2^ = 0.27; *P* = 0.135). When looking at motor performance in detail, we found that a slower attainment of b1-RT was significantly associated with poorer performance on the Movement-ABC balance task (*B* = −0.420; 95% CI, −0.837 to −0.003; *R*^2^ = 0.220; *P* = 0.048; not shown). Adding the interaction term b1-RT non-competitive*group revealed no significant effects.

**Figure 3:**
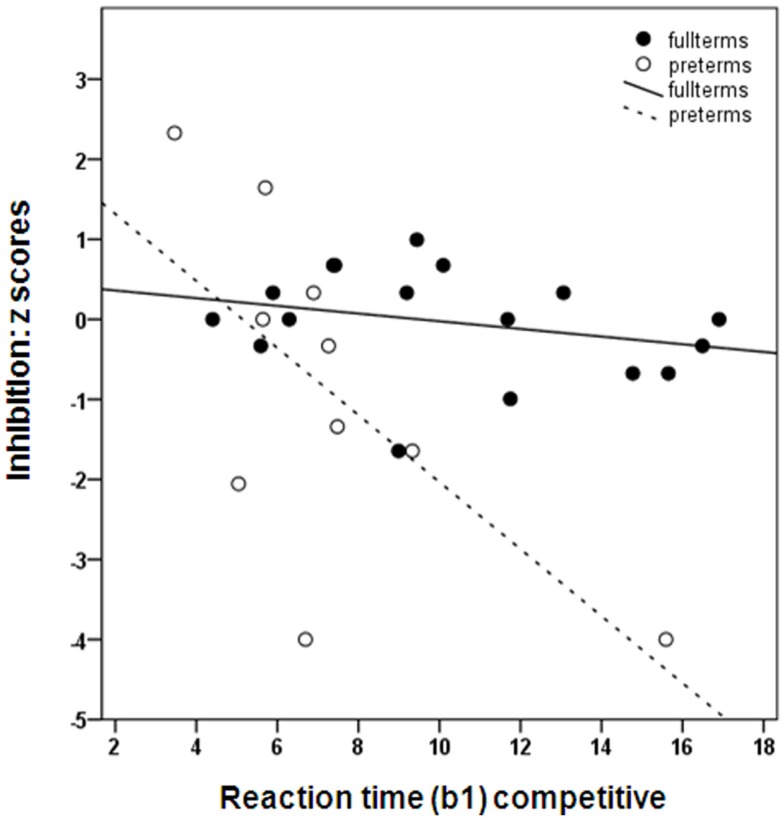
**The speed at which the infant grows toward a lower reaction time (b1) in relation to *z* scores on TEA-Ch-NL opposite world (inhibition)**. A higher b1 value represents a slower development toward the adult level reaction time (200 ms).

Under the competitive conditions, a slower attainment of 50%-looks was marginally associated with poorer handwriting skills at school age (OR = 1.44; 95% CI, 0.950–2.168; *R*^2^ = 0.468; *P* = 0.086; not shown). Adding the interaction term 50%-looks competitive*group revealed no significant effects. Regarding the latencies of looks (b1-RT), we found no significant associations with IQ at school age (*B* = −0.056; 95% CI, −0.130–0.019; *R*^2^ = 0.28; *P* = 0.135). Replacing the composite IQ score by verbal IQ or performance IQ also revealed no significant associations (*B* = −0.051; 95% CI, −0.136–0.034; *R*^2^ = 0.34; *P* = 0.224 and *B* = −0.060; 95% CI, −0.153–0.033; *R*^2^ = 0.12; *P* = 0.197, respectively). We did find that a slower attainment of b1-RT was associated with poorer attention at school age (*B* = −0.102; 95% CI, −0.197 to −0.008; *R*^2^ = 0.37; *P* = 0.035). Of the two attention tasks administered at school age, only the task measuring inhibition of an automatic response showed a significant association (*B* = −0.188; 95% CI, −0.323 to −0.053; *R*^2^ = 0.385; *P* = 0.008). Adding the interaction term b1-RT competitive*group revealed that there was a marginally significant stronger negative effect of slow attainment of b1-RT regarding performance on the inhibition task in preterms than in fullterms (RT competitive *B* = −0.095; 95% CI, −0.254–0.063; *R*^2^ = 0.48; *P* = 0.225 and RT competitive*group *B* = −0.290; 95% CI, −0.585–0.006; *R*^2^ = 0.48; *P* = 0.054; see Figure 3). After applying Bonferroni corrections, none of the predictive associations reached significance.

To summarize, a slower development toward adult latencies under non-competitive conditions predicted poorer performance on the Movement-ABC balance task. A slower development toward adult latencies under competitive conditions predicted poorer inhibitory attentional control at school age. This association was marginally stronger in preterm-born children than in fullterm-born children.

We repeated the analyses with averaged composite *z* scores at school age to investigate whether visual attention markers were predictive of overall functioning at school age. The mean overall composite *z* scores were 0.19 (SD 0.36) for fullterms and −0.58 (SD 0.52) for preterms (*P* < 0.001). Our analyses revealed no significant associations between visual attention markers during the first 6 months and overall functioning at school age, neither under non-competitive conditions (50%-looks *B* = −0.026; 95% CI, −0.076–0.024; *R*^2^ = 0.54; *P* = 0.294 and b1-RT *B* = −0.080; 95% CI, −0.187–0.027; *R*^2^ = 0.57; *P* = 0.134), nor under competitive conditions (50%-looks *B* = −0.024; 95% CI, −0.075–0.028; *R*^2^ = 0.54; *P* = 0.348 and b1-RT *B* = −0.036; 95% CI, −0.080–0.009; *R*^2^ = 0.57; *P* = 0.111). No significant interaction effects with group were found, neither under non-competitive conditions (50%-looks *B* = 0.068; 95% CI, −0.076–0.213; *R*^2^ = 0.56; *P* = 0.336 and b1-RT *B* = 0.138; 95% CI, −0.096–0.372; *R*^2^ = 0.59; *P* = 0.235), nor under competitive conditions (50%-looks *B* = −0.032; 95% CI, −0.135–0.072; *R*^2^ = 0.55; *P* = 0.531 and b1-RT *B* = −0.024; 95% CI, −0.129–0.082; *R*^2^ = 0.58; *P* = 0.648).

## Discussion

In this exploratory study, we investigated whether the developmental course of gaze shifts and latencies toward a peripheral stimulus during the first 6 months postterm were associated with cognitive and motor outcomes at school age. Subsequently, we determined whether predictive associations differed between fullterms and preterms. Compared to fullterms, preterms developed adult gaze shift latencies under competitive conditions faster. At school age, overall performance of preterms, their visuomotor and motor skills in particular, were poorer than that of fullterms. The rate of development of early visual attention was not associated with overall functioning at school age. Nevertheless, some visual attention markers predicted functional difficulties on specific domains. At school age, we found marginal differences in predictive associations for inhibitory attentional control and comprehensive reading.

We first discuss the predictive associations of visual attention measures in infancy with outcomes at school age. Subsequently, we discuss the differences in predictive associations between fullterms and preterms.

Regarding measures under non-competitive conditions, our data indicated that infants whose development of gaze shifts (both looks and latencies) was slower had poorer motor skills at school age, specifically poorer balance. Put differently, infants who developed efficient visuomotor processing more slowly had poorer balance later. A meta-analysis by Wilson and McKenzie (55) concluded that difficulties in visual information processing are common in children diagnosed with developmental coordination disorder (DCD) at preschool age and beyond. This study provided the first data that motor development and attentional development might also be associated longitudinally. We suggest that the cerebellum is involved in this association. Supporting evidence for the role of the cerebellum in both gaze shifts, and reaching and maintaining balance, can be found in patients with cerebellar lesions. Cerebellar lesions, specifically focal lesion in the cerebellar vermis, are known to cause balance impairments (56, 57) as well as abnormalities in the initiation of pursuit eye movements (58, 59). This observation indicates that the cerebellar vermis and the superior colliculus, the key structure in the generation of saccades, may be closely linked. Indeed, several models have been proposed that suggest a close cooperation between the superior colliculus and the cerebellum, including the vermis, during saccadic eye movements (60–62). In addition, the cerebellum is considered an important structure in the acquisition and execution of automatic movements (63). The eye movements and the balance demanding tasks in our study both rely on automatic processing. There appears to be an anatomically commonality in that the cerebellum is considered a central structure in eye movements and balance tasks. At present, however, the precise mechanisms underlying the longitudinal relationship between visuomotor processes in infancy and balance at school age is not properly understood.

Regarding predictive associations of visual measures under competitive conditions, our most prominent finding is that infants who attained adult latencies at later postterm ages, had poorer attention scores at school age. More specifically, their performance on a task that measured inhibition of an automatic response (TEA-Ch-NL Opposite world) was poorer. Stability in cognitive abilities over time has been demonstrated before (34, 64). To date, one other study on preterms found visual attention in early infancy to be predictive of attention at school age. Sigman and colleagues (8) reported that preterms who fixated a single stimulus longer at term age had poorer scores at school age on a novelty test that measured the ability to shift attention while ignoring irrelevant cues. Although these authors used different infant and school age measures of attention, basically their results are in agreement with ours. Infants with longer fixation durations, i.e., infants who had difficulty disengaging from a stimulus, also had poorer inhibition of attention to irrelevant information at school age. Others proposed that the ability to shift the gaze away from repetitive or uninformative aspects of the visual environment may reflect better attentional capabilities due to efficient information processing (3, 65). According to this view, the inability to quickly disengage from a fixated stimulus may in turn reflect poorer attentional capabilities to the detriment of attentional abilities at school age. Rothbart and colleagues (66) proposed that exercising the orienting network by presenting novel objects may produce increased connectivity between parietal areas involved in the orienting network and the lateral and medial frontal areas. Later on in development, these latter areas are connected to the executive control network, the attention network involved in resolving conflict among response tendencies (67). Although the executive network is not yet fully operational before the age of 3–4 years (66), a strong functional connectivity between the orienting and executive networks is already in place during the first 2 years after birth (68). Inability to quickly disengage one’s gaze may decrease the opportunities of exploring the surrounding visual world and may, as a consequence, lead to decreased connectivity in lateral and medial frontal areas later connected to the executive network. Our findings indicated that the rate at which the ability to disengage under competitive conditions developed during the first 6 months, may serve as a critical component for later inhibitory attention control, possibly by a mechanism involving complex cortical–subcortical circuits.

Some findings applied to both non-competitive and competitive conditions. For instance, we found that those children who had been slower in developing looks under non-competitive and competitive conditions as infants, had a poorer handwriting at school age, a skill that requires visuomotor integration. We were, however, unable to replicate this finding with the visuomotor integration task (NEPSY-II Design copying). Further study is needed to clarify the broader significance of this finding.

We were unable to demonstrate significant associations between visual attention measures in infancy and IQ at school age. This is in contrast to previous research (7, 33–35). These researches all suggested early visual attention measures to be predictors of IQ at school age. A possible explanation why such an association was absent in our study might be related to the age at which infants were tested. In the studies mentioned, infant measures of visual attention were taken from the age of 7 months onward, whereas we associated the developmental course of visual attention during the first 6 months to later functioning. A hypothetical explanation could be that the mental abilities that determine later intelligence are not yet sufficiently stable during the first months postterm due to the relative immaturity of the brain at this time (69). Moreover, the previous studies used static stimuli (abstract stimuli or neutral faces). Instead, we used dynamic stimuli, either abstract or representing the mother’s face. This might have facilitated shifting the gaze toward the peripheral stimulus (70–72).

Our second research objective was to determine whether predictive associations differed between fullterms and preterms. Apart from the predictive relations it became clear that preterm infants performed below their fullterm-born peers on visuomotor skills, motor skills, and executive functioning, which is in accord with findings from a large cohort study of very preterm infants (73). In the preterm group, we found a tendency for slower attainment of 50%-looks under non-competitive conditions to be associated with better scores on comprehensive reading as opposed to poorer scores for the fullterms. Our findings imply that too rapid a development of visuomotor processes due to preterm exposure to the visual world might be disadvantageous for comprehensive reading later on. This is in line with our expectations. We assumed the faster development in preterms to occur at the expense of the formation of stable neural networks, meaning that slower development would be predictive of better, rather than poorer, functioning at school age. Even so, we reasoned that in fullterms more rapid development of visual attention may be a sign of maturity and therefore associated with better functioning at school age.

With regard to differences in predictive associations under competitive conditions, we found no opposing associations for fullterms and preterms. Nevertheless, we did find that the negative relationship between the development of gaze shifting latencies under competitive conditions and later inhibitory attentional control was more pronounced in preterms than in fullterms. Important to note is that under the competitive conditions the preterms reached adult latencies of looks at an earlier postterm age than fullterms, indicating an advanced development of cortical processing in preterms (25). Despite their overall faster development of attentional disengagement, the preterms who seemed to have benefited least from their additional visual exposure had the lowest attention scores at school age (see Figure 3). Presumably, early visual attention is a more robust marker of later attention skills in preterm-born than in fullterm-born children. This replicates findings from previous studies that predictive associations with later functioning are stronger in risk samples than in non-risk samples (33, 64, 74, 75). The stronger predictive values for the preterms may also be a chance finding due to the small sample size and the presence of more extreme scores in the preterm group compared to the fullterm group.

In general, preterm or fullterm birth and the level of maternal education were related to most of the performance measures at school age. Predictive associations, however, did not differ between fullterm-born and preterm-born children. Our findings are in line with Rose and colleagues who found no indications of differences between fullterms and low-risk preterms in the predictive relationships of several infant measures for IQ up to 11 years (33, 34, 76). We now add that this might also hold true for several other domains. Apparently, early visual exposure in preterms does not have much impact on the formation of neural networks involved in later cognitive and motor performance.

This study was one of the first to undertake a detailed exploration of the associations between visual attention in infancy and cognitive and motor outcomes at school age using standardized, skill-based measures. Nevertheless, we did also encounter some limitations. First, our study group was small. We may therefore have missed true associations due to a lack of power. Additionally, the chosen cut-off *P* value of 0.15 for analyzing subscales of composite scores might also have caused an underestimation of true associations. Conversely, we should consider the likelihood of Type-I errors in view of the large number of comparisons. We attempted to limit the number of chance findings by only analyzing subscales of composite scores when *P* values reached levels below 0.15. In addition, we re-evaluated our results after applying Bonferroni adjustments. A potential criticism, however, is that such statistical adjustment might have been too conservative, in particular because both the visual attention measures and the school age outcome scores might be correlated. Second, our fullterm sample may not be fully representative of the general population in the sense that most mothers had a relatively high level of education. Finally, we emphasize that all the predictive associations and their potentially underlying neural substrates as elaborated on in the discussion should be interpreted cautiously because after correcting for multiple testing none of our findings remained significant.

In conclusion, our findings suggest that a slower developmental course of visual attention measures during the first 6 months postterm age is associated with poorer school age performance on specific domains, irrespective of fullterm, or preterm birth. In particular, a slower development of visuomotor processes predicted poorer balance, whereas a slower development of attentional processes predicted poorer inhibitory attentional control. Our data indicated no clear advantage or disadvantage for preterm-born children of the extra visual exposure in infancy for cognitive and motor outcomes at school age. We speculate that additional visual exposure might not interfere with the ongoing development of related neuronal networks. Our study, however, needs to be understood as an exploratory study, which attempts to begin to address the question whether early visual attention is related to future outcome. For definite answers, we recommend this study be replicated in larger samples of fullterms and preterms. A better understanding of the relationships between markers of early visual attention and later cognitive and motor development may aid both early identification of risk factors and adequate intervention. Our results may serve as a framework for further exploration.

## Conflict of Interest Statement

The authors declare that the research was conducted in the absence of any commercial or financial relationships that could be construed as a potential conflict of interest.

## Supplementary Material

The Supplementary Material for this article can be found online at http://www.frontiersin.org/Journal/10.3389/fped.2014.00106/abstract

## Footnotes

^1^For a detailed report on the development of gaze shifting during the first 6 months of life in the preterm and fullterm group, see Hunnius et al. (25). Please note that 3 fullterm children from the initial sample had to be excluded because there were no follow-up data available.
