# Supplementary material for: Early Visual Attention in Preterm and Fullterm Infants in Relation to Cognitive and Motor Outcomes at School Age: An Exploratory Study
Source: Front Pediatr. 2014 Oct 6;2:106. doi: 10.3389/fped.2014.00106 (PMC4186265; doi:10.3389/fped.2014.00106)
Supplement: Supplementary file 1 [file Data_Sheet1.DOCX]

**Supplement**

Table. Cognitive and motor outcomes of the fullterm and preterm children

|  | Fullterms | Preterms | *P* value |
| --- | --- | --- | --- |
| *Cognitive outcomes* |  |  |  |
| Total intelligence^a^ | 101 ± 10 | 96 ± 13 | .249 |
| Verbal intelligence^a^ | 108 ± 13 | 101 ± 12 | .191 |
| Performance intelligence^a^ | 94 ± 12 | 91 ±14 | .506 |
| Selective attention^b^ | 57 (0.1−91) | 37 (5−75) | .182 |
| Inhibition^b^ | 50 (5−84) | 23 (0.1−99) | .223 |
| Visuomotor integration^b^ | 26 (11−51) | 19 (2−26) | .009** |
| Picture puzzles^b^ | 51 (11−76) | 19 (11−76) | .040* |
| Arrows^b^ | 91 (37−100) | 63 (16−99) | .127 |
| Route finding^b^ | 26 (26−51) | 26 (0−26) | .309 |
| Form constancy^b^ | 84 (25−99) | 38 (1−91) | .027* |
| Visual closure^b^ | 84 (25−99) | 37 (5−91) | .027* |
| Visual discrimination^b^ | 75 (16−99) | 31 (9−99) | .016* |
| Executive functioning^c^ | 11 (1−88) | 61 (13−90) | .013* |
| Mathematics (Levels I, II or III)^d^ | 15 (88) | 6 (60) | .153 |
| Spelling (Levels I, II or III)^d^ | 16 (94) | 5 (50) | .015* |
| Comprehensive reading (Levels I, II or III)^d^ | 14 (82) | 6 (60) | .365 |
| Technical reading (Levels I, II or III)^d^ | 12 (71) | 6 (60) | .673 |
| *Motor outcomes* |  |  |  |
| Movement-ABC total^e^ | 10.0 (1.5−26.0) | 11.3 (7.0−24.0) | .127 |
| Manual dexterity^e^ | 3.5 (1.0−7.5) | 7.5 (3.0−10.0) | .005** |
| Object control^e^ | 4.5 (0.0−8.0) | 3.0 (0.0−6.0) | .443 |
| Postural control^e^ | 0.5 (0.0−11.0) | 3.0 (1.0−10.0) | .066 |
| Handwriting (BHK)^e^ | 14 (4−28) | 22 (12−30) | .002** |
| Motor problems (DCD-Q)^e^ | 70 (15−75) | 63 (29−74) | .339 |

Data are given as median (minimum−maximum), mean ± SD or numbers (percentage). BHK, Beknopte beoordelingsmethode voor Handschriften van Kinderen [Concise Assessment Scale for Children’s Handwriting]; DCD-Q, Developmental Coordination Disorder Questionnaire*. P* values represent statistical differences between the fullterm and preterm group as calculated by the Fisher’s exact test (categorical variables), the Mann-Whitney *U* test or the Student *t* test (continuous variables). **P* < 0.05; ***P* < 0.01.

a. intelligence quotients

b. percentiles (low percentile indicates poor outcome)

c. percentiles (high percentile indicates poor outcome)

d. scaled scores according to Dutch norms (higher levels indicating poor outcome)

e. raw scores
